# *Amiskwia* is a large Cambrian gnathiferan with complex gnathostomulid-like jaws

**DOI:** 10.1038/s42003-019-0388-4

**Published:** 2019-05-03

**Authors:** Jean-Bernard Caron, Brittany Cheung

**Affiliations:** 1 Department of Ecology and Evolutionary Biology, University of Toronto, Toronto, ON M5S 3B2 Canada; 2 Department of Natural History—Palaeobiology, Royal Ontario Museum, 100 Queen’s Park, Toronto, ON M5S 2C6 Canada; 3 Department of Earth Sciences, University of Toronto, Toronto, ON M5S 3B1 Canada

**Keywords:** Palaeontology, Evolutionary theory

## Abstract

Phylogenomic studies have greatly improved our understanding of the animal tree of life but the relationships of many clades remain ambiguous. Here we show that the rare soft-bodied animal *Amiskwia* from the Cambrian of Canada and China, which has variously been considered a chaetognath, a nemertine, allied to molluscs, or a problematica, is related to gnathiferans. New specimens from the Burgess Shale (British Columbia, Canada) preserve a complex pharyngeal jaw apparatus composed of a pair of elements with teeth most similar to gnathostomulids. *Amiskwia* demonstrates that primitive spiralians were large and unsegmented, had a coelom, and were probably active nekto-benthic scavengers or predators. Secondary simplification and miniaturisation events likely occurred in response to shifting ecologies and adaptations to specialised planktonic habitats.

## Abstract (web summary)

Jean-Bernard Caron and Brittany Cheung provide new evidence that the rare Cambrian animal *Amiskwia* is closely related to gnathiferans, thereby resolving its ambiguous phylogeny. They describe new specimens from the Burgess Shale that preserve the complex jaw apparatus, which is most similar to gnathostomulids.

## Introduction

The Burgess Shale yields a treasure trove of fossils dating back to the middle Cambrian period (Wuliuan stage, ca. 505 million years ago). In addition to the usual shelly fauna, this site preserves an exceptionally diverse and abundant community of soft-bodied marine animals, including a number of species with seemingly indeterminate morphologies. Referred to as “problematica”, such taxa have traditionally been difficult to classify within modern groups of animals^1^. However, thanks to continuous progress in our understanding of animal relationships, new fossil discoveries, and the application of modern imaging and analytical techniques for studying fossils, many problematic taxa have become vital in reconstructing the early evolution of bodyplans and the sequence of acquisition of morphological characters that led to modern phyla^2^.

*Amiskwia sagittiformis* remains one of the most enigmatic fossils of the Burgess Shale. It was originally described by Walcott in 1911 as a chaetognath^3^. This interpretation was rejected about half a century later by several authors in favour of a nemertine affinity^4,5^. Conway Morris restudied Walcott’s material in 1977 and concluded that *Amiskwia* could not be aligned with any modern phylum^6^. Since then, the chaetognath hypothesis has been reconsidered based on taphonomic grounds^7,8^ and a more recent reinvestigation of Walcott’s five original specimens^9^ (see discussion). *Amiskwia* has also been briefly compared to molluscs^10^ and to the problematic animal *Vetustovermis*^11^, now regarded as a close relative to the cephalopod-like *Nectocaris*^12^, although in both cases such comparisons were unsupported and remained speculative. A second species, *Amiskwia sinica*, has also been described succinctly based on a single poorly preserved specimen recovered from the lower Cambrian of China^13^. Unfortunately, the few preserved features of this species do not provide any new useful knowledge of the genus^14^. In addition, an attempt to locate the holotype specimen in China in 2018 was unsuccessful. A possible second specimen of *Amiskwia sinica* (contra^15^, see Fig. 3 of that paper) does not show convincing grasping spines or fin rays.

In view of these issues and conflicting interpretations, a modern restudy of the Burgess Shale material in particular was long overdue. This paper critically re-evaluates the morphology and affinities of *Amiskwia sagittiformis* based on Walcott’s original five specimens and 21 previously unpublished specimens collected by the Royal Ontario Museum from the Burgess Shale since 1988. Our results suggest affinities with gnathiferans (as a stem group member or within the crown group), a clade containing some of the smallest animals on Earth, and challenge previous views that the earliest spiralians were meiofaunal acoelomates or pseudocoelomates^16–18^.

## Results

### General anatomy

**Fig. 1:**
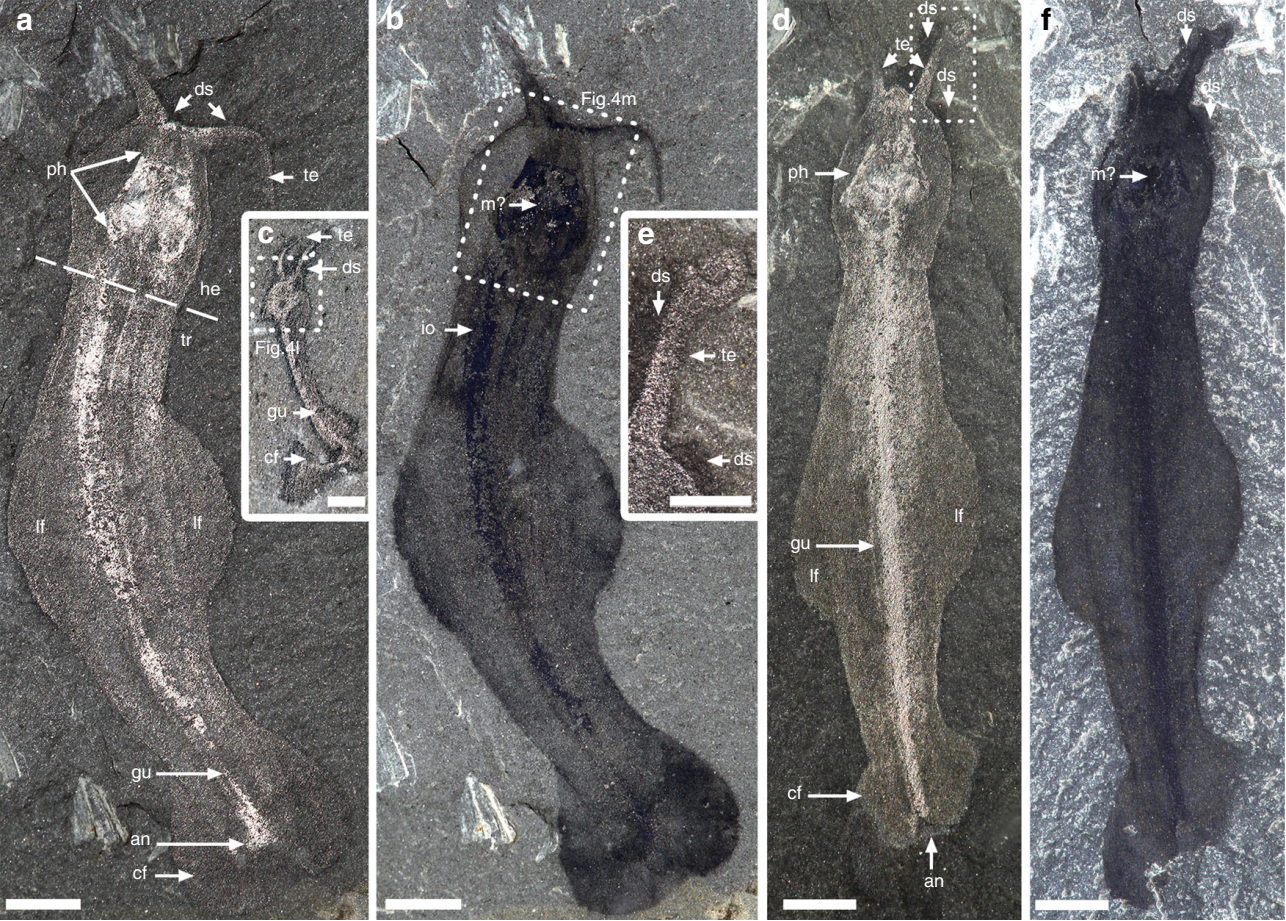
*Amiskwia sagittiformis*—overall morphology. **a**, **b** Lectotype USNM 57644 under direct (**a**) and cross-polarised light (**b**). **c** ROMIP 65047. **d**–**f** Paralectotype USNM 57645 under direct (**d**) and cross-polarised light (**f**). **d**, **f** Full specimen. **e** Close-up of tentacle tip. Scale bars, 1 mm (**c**, **e**), and 2 mm (**a**, **b**, **d**, **f**). an, anus; cf, caudal fin; ds, dark stain; gu, gut; he, head; io, indeterminate organ; lf, lateral fin; m?, mouth?; ph, pharyngeal jaw apparatus; te, tentacles; tr, trunk

**Fig. 2:**
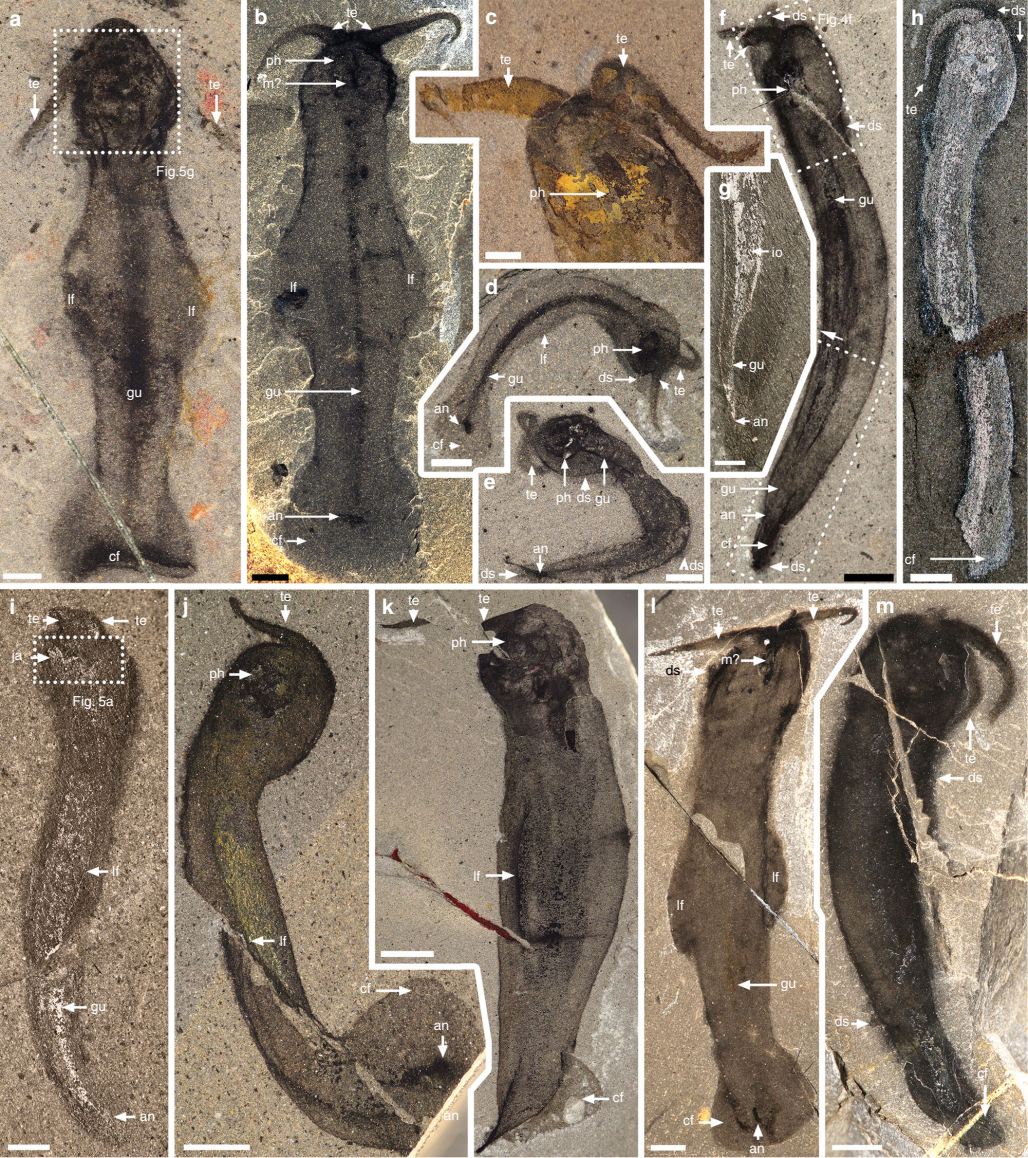
*Amiskwia sagittiformis*—orientations. **a**–**e, l** Dorso-ventral. **a** ROMIP 65046, **b** ROMIP 61122. **c** ROMIP 65035. **d** ROMIP 64015. **e** ROMIP 65042. **l** ROMIP 65030. **f**–**k**, **m** Lateral specimens, ventral side to the left, except **m. f, g** ROMIP 64014. **f** Full specimen. **g** Close-up of posterior region. **h** ROMIP 65036. **i** ROMIP 65038. **j** ROMIP 65041. **k** ROMIP 65033. **m** ROMIP 65044. Scale bars, 1 mm (**a**, **c**, **d**, **e**, **f**, **g**, **h**, **i**), 2 mm (**b**, **f**, **j**, **k**, **l**, **m**). an, anus; cf, caudal fin; ds, dark stain; gu, gut; io, indeterminate organ; ja, jaws; lf, lateral fin; m?, mouth?; ph, pharyngeal jaw apparatus; te, tentacles

**Fig. 3:**
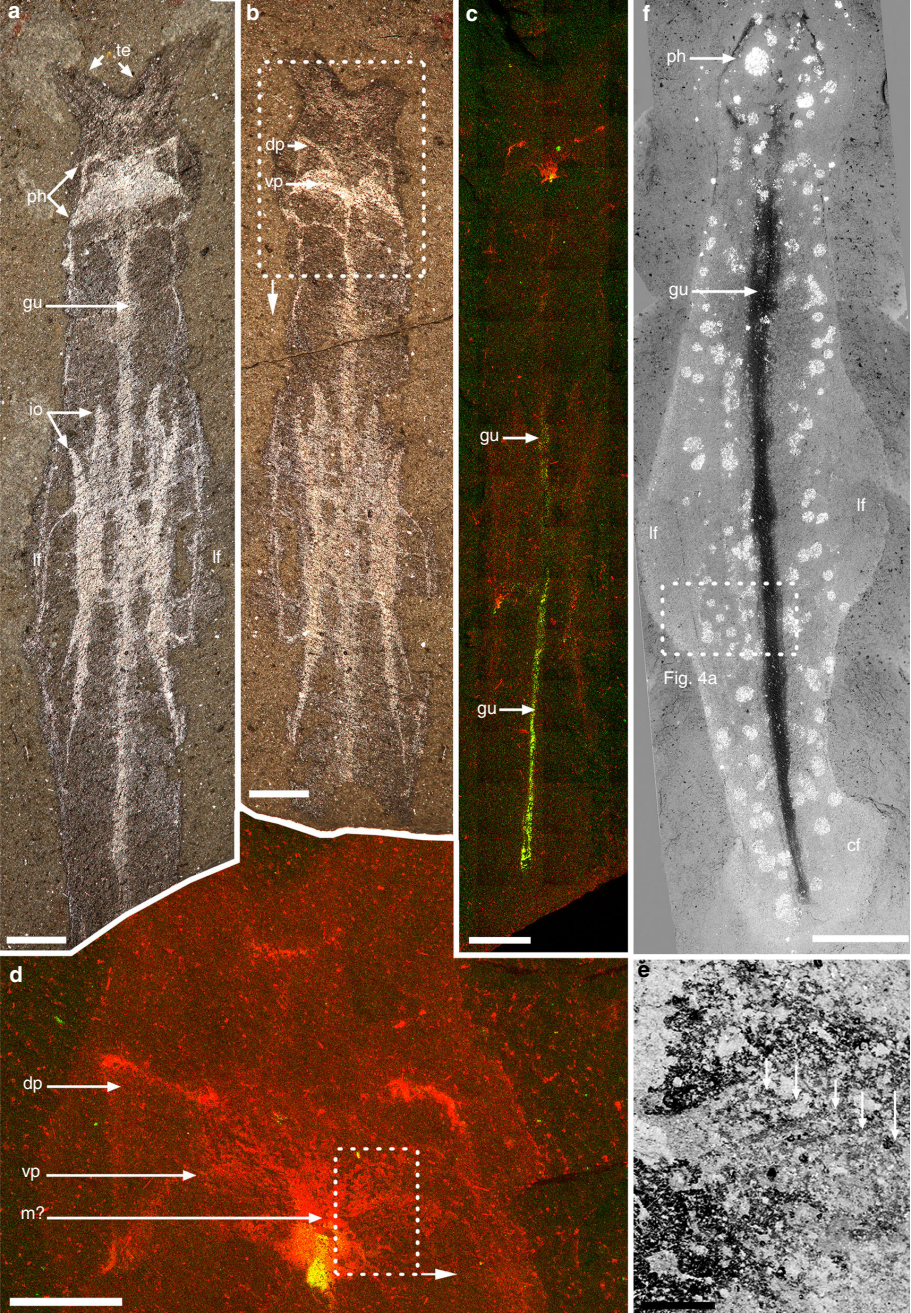
*Amiskwia sagittiformis*—internal anatomy. **a**, **b**, **c**, **d**, **e** ROMIP 64013. **a**, **b** Part and counterpart of full specimen. **c**, **d** Elemental map showing enrichment in carbon (red) and phosphorus (green), full specimen (**c**) and close-up of head (**d**). **e** Close-up of pharyngeal area showing striations on ventral plate (arrows). **f** Paralectotype USNM 57645, full specimen. Elemental maps (**c**, **d**), backscattered electron microscopy images (**e**, **f**). Scale bars, 2 mm (**a**, **b**, **c**, **f**), 1 mm (**d**) and 200 μm (**e**). dp, dorsal plate; gu, gut; lf, lateral fin; m?, mouth?; ph, pharyngeal jaw apparatus; vp, ventral plate

**Fig. 4:**
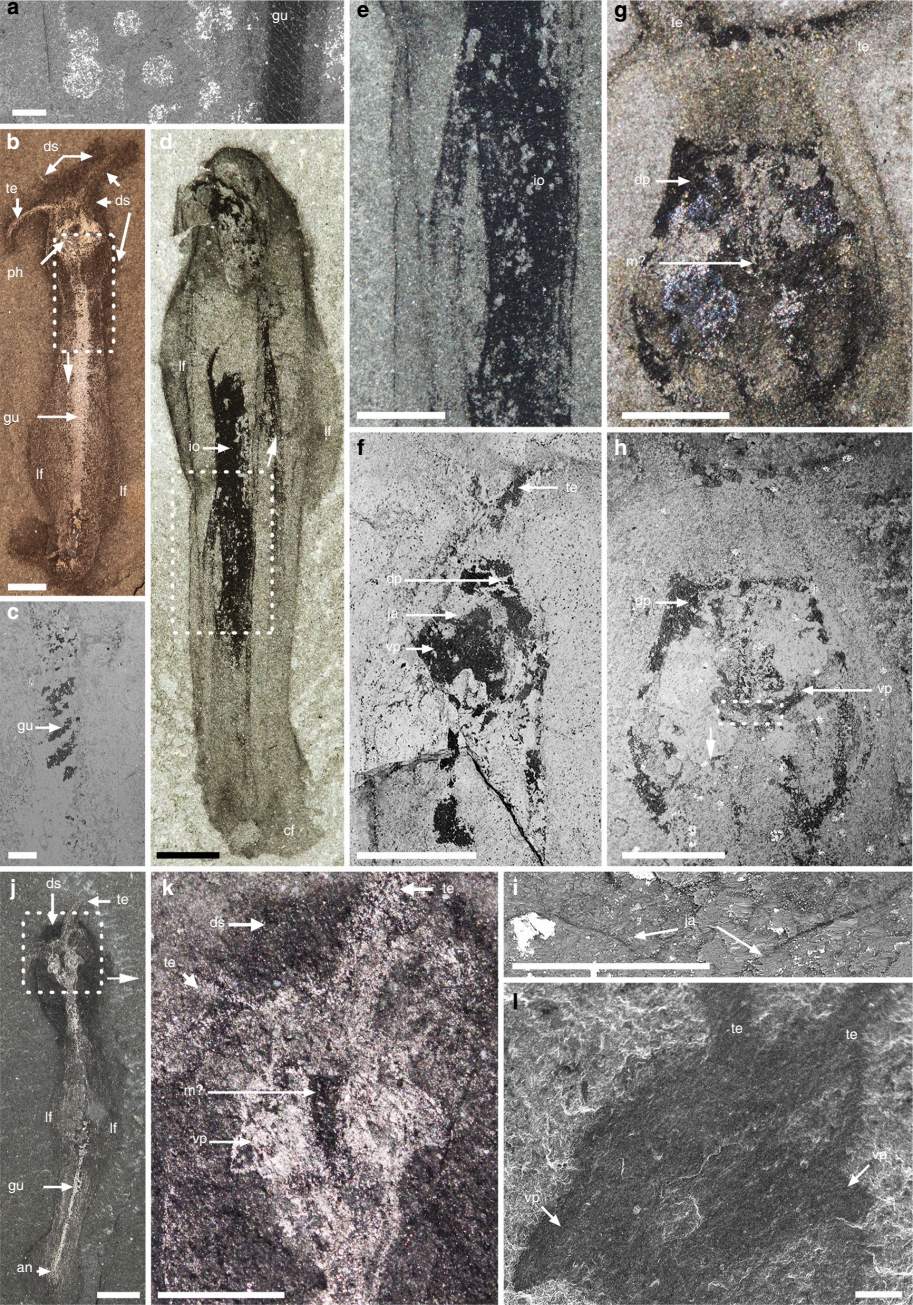
*Amiskwia sagittiformis*—Pharyngeal area and details of internal organs. **a** Paralectotype USNM 57645, close-up of trunk showing tissue underneath the epidermis and surrounding the gut. **b**, **c** ROMIP 64016. **b** Full specimen. **c** Close-up showing anterior gut looping. **d**, **e** USNM 203022. **d** Full specimen. **e** Close-up of dark tissue bundles. **f** ROMIP 64014, close-up of pharyngeal area in lateral view. **g**, **h**, **i** Lectotype USNM 57644. **g**, **h** Close-up of head. **i** Close-up showing possible remnants of the jaws. **j**, **k** Paralectotype USNM 198670. **j** Full specimen. **k** Close-up of head. **l** ROMIP 65047, close-up of head showing dissociated ventral plates extending beyond body outline. Backscattered electron microscopy images (**a**, **c**, **f**, **h**, **i**), or secondary electron microscopy image (**l**). Scale bars, 200 μm (**a**, **c**, **i**, **l**), 1 mm (**b**, **e**, **f**, **g**, **h**, **k**), and 2 mm (**d**, **j**). an, anus; dp, dorsal plate; ds, dark stain; gu, gut; io, indeterminate organ; ja, jaws; lf, lateral fin; m?, mouth?; te, tentacles; vp, ventral plate

*Amiskwia* has a conspicuous head with tentacles and an elongate trunk about two thirds of the total body length. The entire animal was evidently flexible, as demonstrated by many specimens (Figs 1c, e and 2a–e, j). Decay was quite frequent, as evidenced by dark stains surrounding the specimens, particularly around the head and tentacles (i.e., Fig. 1a, c, d, f), tattered fins (Figs 2k and 3a, b), and blurring of internal features (i.e., Figs 1d and 2a, b). Specimens vary from 7.4 to 31.3 mm in length (average = 18.5 mm, *n* = 22), excluding the tentacles, and from 0.5 to 5.5 mm in width (average = 2.8 mm, *n* = 18), measured along the mid-trunk, excluding the lateral fins. When viewed laterally (Fig. 2f–k, m), the body is about one third narrower than it is wide, confirming that it was dorso-ventrally flattened^6^. The head is somewhat flatter ventrally and more rounded dorsally (Fig. 2f, h, j, m) with the tentacles arising near the anterior midline, projecting to the front and sideways (Figs 1, 2a–e, j, 3a, b and 4b). The tentacles have a thick base and taper to a thin point, often curling near their tips (Figs 1d–e and 2c, e). The trunk gently tapers toward the caudal fin and is wider near the middle, where it supports the lateral fins, which represent around one third of the body length (e.g., Fig. 1a, d). The caudal fin, which is roughly one fifth of the body length, is flat, round in outline, and completely surrounds the posterior trunk (e.g., Figs 1a and 2b, l). Both lateral and caudal fins are inserted along the same horizontal plane (Fig. 2k). The fins and the rest of the body preserve in similar ways and there is no evidence of fin rays^6^.

### Internal organs and pharyngeal jaw apparatus

Internal structures include a partially phosphatized gut (Fig. 3c), which runs from a ventrally positioned mouth to the ventral anus, below the anterior section of the caudal fin (Figs 1a, b, d, f, 2b, d–g, i, j, l and 4j). The gut is straight, varies in thickness, and might spiral near the anterior section (Fig. 4c). Within the head is a large, highly reflective quadrate area (e.g., Fig. 1a). This structure is more or less parallel to the ovoid outline of the head and retains a similar shape in most specimens studied, suggesting it was relatively rigid, which is also supported by its preservation as a thick layer of carbon (Fig. 3d). The quadrate area is roughly half the size of the head in dorsal view but is flatter in lateral view (Figs 2f, h, j and 4f). A depression in the middle where the gut terminates coincides with the likely position of the mouth (e.g., Figs 2j, l 3d and 4g, k).

**Fig. 5:**
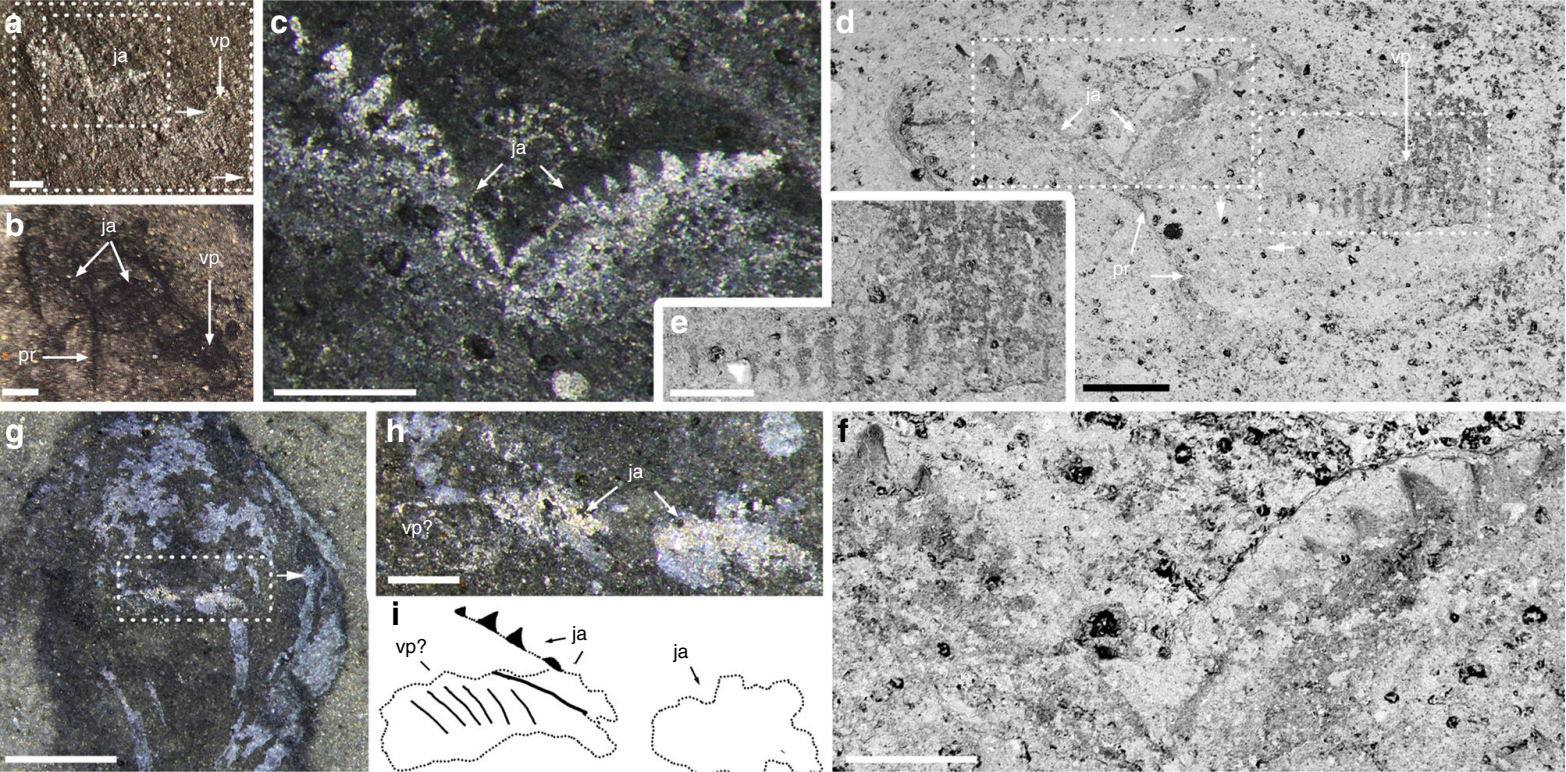
*Amiskwia sagittiformis*—jaw morphology. **a**, **b**, **c**, **d**, **e**, **f** ROMIP 65038 (Fig. 2i, full specimen). **a**, **b** Close-up of pharyngeal area under direct (**a**) and cross-polarised light (**b**). **c** Close-up of jaws. **d** Larger close-up of area in **a**. **e** Close-up of grooves or raised areas, presumably on ventral plate. **f** Close-up of teeth. **g**, **h**, **i** ROMIP 65046 (Fig. 2a, full specimen). **g** Close-up of head. **h**, **i** Close-up of jaws and ventral plates (**h**) and interpretative drawing (**i**). Backscattered electron microscopy images (**d**, **e**, **f**). Scale bars, 100 μm (**e**, **f**), 200 μm (**a**, **b**, **c**, **d**, **h**) and 1 mm (**g**). ja, jaws; pr, posterior rod; vp, ventral plate

**Fig. 6:**
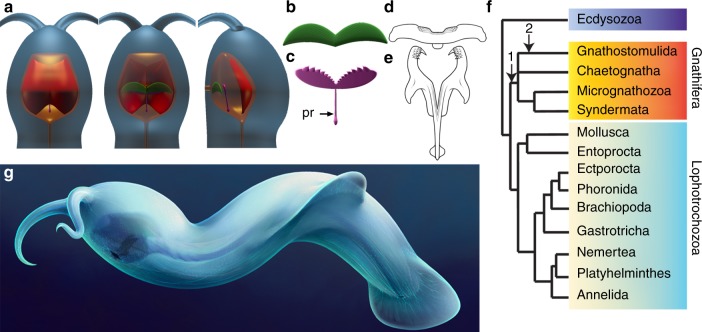
Reconstructions and phylogenetic relationships of *Amiskwia sagittiformis*. **a** Dorsal (left), ventral (middle) and lateral (right) views of the head. **b** Ventral plate in dorsal view. **c** Jaws in ventral view. **d**, **e** Drawings of basal plate and jaw of extant Filospermoidea gnathostomulids; **d** *Cosmognathia aquila*, **e** *Haplognathia gubbarnorum*. **f** cladogram based on ref. ^23^, showing possible phylogenetic positions of *A. sagittiformis* within Gnathifera: stem gnathiferans (1), stem gnathostomulids (2). **g** Artistic reconstruction. Figure panels (**a**–**e**, **g**) by Brittany Cheung © Royal Ontario Museum

Behind the mouth is a pair of semi-circular elements, which occupies about two thirds of the width of the quadrate area. Each of these elements, which we called jaws, bears about eight to ten stout conical teeth, which increase in size laterally and project forward (Fig. 5c, d, f–i). Preserved in a butterfly position, the jaws are seemingly connected axially and posteriorly by a single elongate rod-like structure with a terminal bulbous section (Fig. 5a, b, d). The rarity of specimens with preserved teeth, evident in only two specimens, is puzzling considering that other tooth-like elements comparable in size and preservation, such as the denticles on the radula of the primitive mollusc *Odontogriphus*, are clearly visible in most specimens^19^. The orientation of the teeth themselves could be a factor; a V-shaped structure, which is sometimes preserved (Fig. 4i), might represent a partial outline of the jaws with the teeth buried at an angle along a different bedding plane. In addition, the jaws are effectively concealed at most angles by dorsal and ventral elements (see reconstruction Fig. 6a–c). One of these structures slightly posterior to the mouth, is a bilobed element with distinct grooves or raised areas radiating antero-laterally from the midline (Fig. 3d, e). Roughly 1.25 times the width of the jaws and presumably ventral to it (Fig. 6a), these plates maintain a clear outline beyond the body outline in dissociated specimens, suggesting they were relatively robust (Figs 1c and 4l).

A third type of structure, roughly hexagonal in shape and presumably dorsal to the jaws, encompasses most of the jaw apparatus (Fig. 6a). This structure appears bilaterally symmetrical with a thickened outline of carbon; the posterior margins are wide and rounded (“oval spaces” of Walcott^3^) whereas the anterior margins are small and pointed (e.g., Figs 1a, d, 3a, b and 4b). The style of preservation is similar to the jaws and the ventral plate, suggesting this structure is part of the jaw apparatus instead of being testes^5^ or a pair of cerebral ganglia with a central commissure^6^. Interpretations of other cephalic internal structures such as nerve cords or blood vessels^6^ remain equivocal.

A proboscis^5^ cannot be identified, as previously discussed^6^, making a nemertean affinity unlikely. Reflective or dark bundles of tissues of unknown identity run on either side of the gut (Figs 1a, 2f, 3a, b and 4d, e).

## Discussion

Hard jaws, i.e., any rigid articulated structures around the mouth used to obtain and process food, have evolved multiple times in animals, often with distinct types in different bodyplans^20^. The preserved morphology of the jaw apparatus in *Amiskwia* is, therefore, of particular phylogenetic significance and should be compared with spiralian taxa that have evolved jaws. The molluscan radula with serially repeated teeth on a radular membrane, a configuration already known in Cambrian forms^19^, is too dissimilar to support a molluscan affinity. The forceps-like jaw elements are somewhat comparable to the main jaws known in some derived annelid polychaetes (Phyllodocida, Eunicida and Ampharetidae)^21^. Closer comparisons with polychaetes are problematic however, since *Amiskwia* does not possess hallmark polychaete characteristics such as segmentation and chaetae, and the evolution of jaws is likely secondary in this group, not appearing at least until the late Cambrian period^22^. Possession of pharyngeal jaws and associated plates of similar complexity to *Amiskwia* are particularly similar to gnathostomulids (Fig. 6b–e), which probably represent the earliest divergent extant gnathiferans, a clade of miniaturized animals which also includes micrognathozoans and rotifers^23^. The forceps-like jaws are connected posteriorly by an elongate structure similar to the symphysis in gnathostomulids and micrognathozoans and the fulcrum in rotifers. Such structures are considered homologous and probably evolved early in gnathiferans^24^. Other parts of the feeding apparatus are more difficult to interpret. The large dorsal plate of *Amiskwia* does not have clear equivalence to pharyngeal structures in extant gnathiferans, although it is possibly homologous to the manubrium of rotifers, which also tends to laterally and dorsally cover large portions of the feeding apparatus^25^. More speculatively, this structure might have had a role in reinforcing the roof of the pharyngeal cavity, although this would make *Amikswia* unique within gnathiferans. The ventral plate in *Amiskwia* could, however, be homologous to the basal plate found in gnathostomulids^26^. Although it does not have teeth at the front as do many extant gnathostomulids, perhaps the striations had a role in food manipulation.

Despite continuing uncertainty related to the position of chaetognaths^27^, a possible connection with gnathiferans has found increasing molecular^23,28–30^ and fossil support^15^. Although disputed^4–6^, a chaetognath affinity for *Amiskwia* has long been proposed based on morphological^3^ and taphonomic grounds^7,8^.

While our paper was under revision, Vinther and Parry^9^ published a concurrent restudy of the five Walcott *Amiskwia sagittiformis* specimens. Vinther and Parry^9^ argue for a close relationship to chaetognaths. What they interpret as a bilateral jaw apparatus corresponds to what we interpret as a pair of ventral pharyngeal plates, although Vinther and Parry^9^ failed to acknowledge that the interpretation of the large reflective area as a feeding apparatus composed of at least one pair of large elements had already been presented by us at a conference in 2016^31^. In our view, Vinther and Parry’s^9^ study not only does not provide any new convincing evidence in support of a chaetognath affinity compared to what has been argued before (see ref. ^6^) but we argue also introduces several misinterpretations, for example, the presence of a putative cephalic hood. A cuticularized cephalic hood is present in modern chaetognaths and perhaps also in the Burgess Shale chaetognath *Capinatator*^32^ (though the evidence for this is rather limited). No hood is present in any of the *Amiskwia* specimens observed in either the Walcott or ROM collections. We argue that what Vinther and Parry^9^ interpret as a hood, for example in USNM 189670 (Fig. 1 in their papers^9^), is clearly a diffused area preserved as a dark stain commonly found in a number of Burgess Shale fossils, and which probably represents preservation of decay fluids. This dark stain extends well beyond the head and tentacles in USNM 189670 and is common in many other *Amiskwia* specimens (i.e., Figs 1e and 2h). Some specimens do show a slight crease between the head and trunk area (i.e., Fig. 1d) along the outline of the body, but we interpret this as a physical consequence of flexures of soft tissues around the narrower post-cephalic area. We see no evidence that this corresponds to a hood structure; none of our light photography images or scanning electron microscopy images show a line of organic tissue crossing this area. Additionally, *Amiskwia’s* head is preserved in the same manner as the body, suggesting that it was not covered by a cuticularized hood. We feel that Vinther and Parry’s interpretative drawings of a cephalic structure are particularly misleading in this regard (Fig. 2c, h, k in their papers^9^).

A second point of contention relates to the fins. It has long been recognised that fins are a feature that have evolved in numerous groups, not just chaetognaths, and are therefore convergent^6^. Vinther and Parry’s^9^ evidence for fin rays is dubious and relies on a single specimen (USNM 57644, Fig. 2l, m in their papers^9^) and fewer visualisation techniques. Contrary to their results and to chaetognaths, we fail to recognise fin rays in any specimens, including this particular one, using both light photography and scanning electron microscopy techniques (Figs 1d and 3f). Other aspects of their study also appear to be erroneous, for example, the authors claim that chaetognaths and rotifers share a dorsal anus; in chaetognaths, the anus is in fact ventral. This and other issues pointed out above challenge the strength of their arguments, particularly the validity of their phylogenetic results. Compelling arguments for linking *Amiskwia* to gnathiferans exist, but the Walcott material told only part of the story. New specimens were critical in this regard. As is typical in Burgess Shale research, more specimens provide a better view of anatomical details and taphonomic variations.

 Possession of cephalic tentacles and lack of grasping spines are two important features that distinguish Amiskwia from Recent chaetognaths and fossil chaetognaths from both the Burgess Shale and Chengjiang^10,32,33^. Other arguments for rejecting a chaetognath affinity have included the position of the anus and lack of body septa^4–6^. However, the septa, if present, were not preserved in any Cambrian chaetognaths^10,32,33^, although this could be due to the rarity of complete body fossils available, and the position of the anus appears quite posterior in *Capinatator*^32^, similar to *Amiskwia* and contrary to modern forms, which have the anus anterior to a septum separating the tail section from the trunk. This suggests that a complete gut and a subterminal anus—likely originally ventral, as demonstrated by *Amiskwia*—might be plesiomorphic in chaetognaths + gnathiferans. In *Amiskwia*, fins representing body extensions are preserved in nearly all specimens. The lack of fin rays is a departure from the condition known in extant chaetognaths^6^, however, fin rays are not preserved in Cambrian chaetognaths, either^32^. This could again be due to the rarity of complete specimens recovered or it could mean that primitive chaetognaths did not have fin rays. While the fins remain broadly similar in shape and position, chaetognath fins are epidermal, thus suggesting a different mode of construction, and thus convergence^6^. A closer relationship to chaetognaths is also problematic on the basis of the pharyngeal jaw apparatus in *Amiskwia*, which clearly differs from the chaetognath condition of having external grasping spines, which were already present in Cambrian forms^15^. Rejecting a close relationship with chaetognaths, *Amiskwia* might instead represent a stem group gnathostomulid or a stem group gnathiferan inclusive of chaetognaths (Fig. 6f). Considering that only a few morphological characters unify extant gnathiferans and so few characters are available in the fossils, resolving the exact position of *Amiskwia* will require further fossil discoveries, as well as progress in molecular phylogenies to better constrain the position of chaetognaths and other gnathiferans. Although chaetognaths are retrieved within gnathiferans in the most recent molecular phylogeny, the internal relationships within this group remain unresolved^23^.

Regardless of the exact position of *Amiskwia* and contrary to some recent suggestions of a miniaturised spiralian acoelomate ancestor^17,18^, any of the above scenarios would imply a number of transformations, including secondary miniaturisation events (and loss of many potentially phylogenetically significant characters)—known widely in many bilaterian lineages^16^—from a macrofaunal, potentially coelomate ancestor, as already suggested from the study of extant^34^ and fossil^32^ chaetognaths. *Amiskwia* provides yet another remarkable example of the critical role of Burgess Shale-type deposits in providing invaluable direct morphological details of the earliest members of extant bodyplans.

## Methods

### Material

Twenty-six specimens, including 21 new specimens from the collections of the Royal Ontario Museum (ROM) and the five previously known specimens collected by Walcott and curated at the National Museum of Natural History (NMNH, formerly United States National Museum—USNM) were examined (Supplementary Data 1). All specimens come from the Burgess Shale’s Walcott Quarry in Yoho National Park (British Columbia, Canada).

### Preparation and observation techniques

Royal Ontario Museum specimens were prepared mechanically, when necessary, to expose parts buried within the matrix, using a micro-engraving tool equipped with a carbide bit. Specimens were observed using a Leica M205C stereomicroscope and photographed using a Canon SLR 5DS R camera under different illuminations, including cross-polarised light and under wet and dry conditions. A Zeiss EVO MA15 scanning electron microscope (SEM) was used to study the Walcott specimens at the NMNH. ROM specimens were studied using an FEI Quanta 200 FEG SEM at the University of Windsor and elemental maps were produced using an energy scanning spectroscopy (EDS) X-ray detector and octane plus silicon drift detector (SDD).

### Reporting summary

Further information on experimental design is available in the Nature Research Reporting Summary linked to this article.

## Supplementary information

Reporting Summary

Description of Supplementary Data

Supplementary Data 1

## Data Availability

All relevant data are available from the authors. Detailed notes on the specimens are available in Supplementary Data 1.
