# Supplementary material for: Amiskwia is a large Cambrian gnathiferan with complex gnathostomulid-like jaws
Source: Commun Biol. 2019 May 3;2:164. doi: 10.1038/s42003-019-0388-4 (PMC6499802; doi:10.1038/s42003-019-0388-4)
Supplement: Supplementary file 2 — Description of Supplementary Data [file 42003_2019_388_MOESM2_ESM.docx]

**Description of Additional Supplementary Files**

**File Name**: Supplementary Data 1

**Description**: Table of specimens.
